# Older adults’ preferences in the utilization of digital health and social services: a qualitative analysis of responses to open-ended questions

**DOI:** 10.1186/s12913-024-11564-1

**Published:** 2024-10-04

**Authors:** Elina Laukka, Sanna Lakoma, Marja Harjumaa, Suvi Hiltunen, Henna Härkönen, Miia Jansson, Riikka-Leena Leskelä, Susanna Martikainen, Paula Pennanen, Anastasiya Verho, Paulus Torkki

**Affiliations:** 1 Faculty of Medicine, Department of Public Health, University of Helsinki, P.O. BOX 00020, Helsinki, 00014 Finland; 2 Nordic Healthcare Group, Vattuniemenranta 2, Helsinki, 00210 Finland; 3 Research Unit of Oulu Advanced Research on Service and Information Systems, University of Oulu, P.O. BOX 8000, Oulu, FI-90014 Finland; 4 Union for Senior Services, Helsinki, 00500 Finland; 5 Research Unit of Health Sciences and Technology (HST), University of Oulu, P.O. BOX 8000, Oulu, FI-90014 Finland; 6 RMIT University, GPO Box 2476, Melbourne, VIC 3001 Australia; 7 Medical Research Center Oulu, Oulu University Hospital and University of Oulu, Oulu, Finland; 8 Faculty of Social Sciences and Business Studies, Department of Health and Social Management, University of Eastern Finland, Kuopio, Finland; 9 School of Wellbeing and Culture, Oulu University of Applied Sciences, P.O. BOX 222, 90101 Oulu, Finland

**Keywords:** Digital technology, Telemedicine, Delivery of healthcare, Social welfare, Elderly

## Abstract

**Background:**

While digital health and social services offer promising solutions, they often overlook the perspectives and needs of older adults. This study aims to comprehensively investigate the preferences of older adults regarding the use and development of digital health and social services.

**Methods:**

The survey spanned from 19 March to 31 March 2023. The study population comprised 1100 Finnish individuals aged 75 and over from across Finland. The study used qualitative inductive content analysis to examine the open-ended responses obtained in the survey.

**Results:**

We identified eight main categories for the older adults’ preferences: usability, service design, and security; training, support, instructions, and information; flexibility of compatible devices; understandable language and interpretation of laboratory results; available and accessible services; desired functionalities; delivery of information for viewing, and personalization.

**Conclusions:**

Older adults’ involvement in digital services’ strategy development is crucial, emphasizing value co-creation and segmentation while avoiding value co-destruction. Segmenting users and understanding their needs aids in customizing services, improving healthcare provision. Further research should assess the impact of segmentation-based training and digital device provision on older adults’ adoption of digital health and social services.

**Supplementary Information:**

The online version contains supplementary material available at 10.1186/s12913-024-11564-1.

## Background

In an increasingly digitalized world, healthcare and social services are undergoing a profound transformation. The emergence of digital health and social services, from telemedicine to mobile apps, promises to revolutionize the way we access, manage, and monitor our wellbeing [1, 2]. Furthermore, the healthcare and social services workforce is diminishing, and digitalization has been proposed as a strategic response to address the dual challenges of escalating demands for these services and a shortage of personnel [3]. While these technological advancements are expected to offer numerous benefits to individuals of all ages, a critical demographic that cannot be overlooked is that of the older population [4]. The global population is aging at an unprecedented rate, and a substantial proportion of senior citizens require complex healthcare services. For instance, by 2025, 30% or more of the Finnish population will be over 60 years old [5]. Raja et al. [4] identified only 13 studies in their scoping review that explored telehealth and digital developments in society involving individuals aged 75 years and older in European countries. Gathering information from older people about their needs in telehealth interventions and prioritizing their perspectives is crucial for successful implementation [6, 7]. Recently, Finland underwent a major health and social care reform, integrating all social and health services offered for older adults by wellbeing services counties [3]. Due to limited financial and personnel resources, wellbeing services counties have emphasized digital services, aiming to increase their use among the Finnish population [3]. As previous research has indicated potential digital exclusion among older adults [8, 9], it is crucial to explore their experiences with current digital health services in Finland.

Older adults have been less inclined to utilize technology for everyday services and communication than younger cohorts, primarily due to factors such as digital literacy and internet access [10]. Older adults have significantly lower internet usage rates than younger age groups, in terms of both general utilization [11] and health-related engagement [12]. According to Mielonen et al. [13], digital health and social services must be accessible and available to older adults, and adequately based on their needs. Older adults are more likely to have poorer cognitive or physical function than adults aged under 75, and this may affect their usability experiences [14, 15]. Heponiemi et al. [16] emphasize the importance of online services being tailored to the requirements of older adults and those with limited digital literacy. McQuown et al. [17] in turn observed in their study that older adults grappling with chronic illnesses may encounter challenges related to the digital divide. However, some older adults are currently satisfied with digital health and social services and consider them useful [18].

Meeting the healthcare demands of older people is an evolving challenge and understanding their preferences for digital health and social services is integral to providing inclusive and effective care. Especially in public healthcare systems, equality targets pose a challenge the rapid increase of digital health and social services. Digital health and social services emphasize the active role of the clients in value co-creation (VCC), and it has been suggested that they could be examined through a service dominant-logic (SDL) [19, 20]. Yet the roles of customers as value creators in digital health and social services have not received sufficient attention [21]. Value co-destruction (VCD) in particular, the opposite to value co-creation, may cause dissatisfaction and prevent or discourage people from using digital health and social services [22, 23]. For example, designing digital health and social services without the input of older adults might undermine the value of digital services for this demographic. Therefore, this study focused on understanding older adults’ preferences and needs for digital health and social services.

To summarize, understanding older adults’ preferences and needs is crucial, as according to Wildenbos et al. [14] the rate of adoption and usage of digital health solutions among adults is low in comparison to that of the population on average, although older adults tend to need more healthcare and social services [24]. In addition, according to Mantovani and Turnheim [25], older adults in Europe are anticipated to adopt technological advancements to the same extent as younger age groups. However, there appear to be numerous barriers that need to be addressed in order for older adults to utilize and derive benefits from digital services [4].

Finally, the involvement of older adults in the design and development of digital services may facilitate their acceptability [26], and help avoid value co-destruction [22]. As we explore the intersection of aging and technology in health and social care, we aim to shed light on how digital health and social services can be designed and implemented to better accommodate the unique needs and expectations of the older population. By considering their perspectives and addressing their preferences, we can pave the way for a more equitable and accessible healthcare and social welfare landscape that benefits individuals of all ages. The objective of the study was to comprehensively investigate the preferences and needs of older adults regarding the use and development of digital health and social services. The research question was:

How can digital health and social services be designed to more effectively meet the needs of the older adults?

## Methods

### Study design

The research was conducted as a survey study, and we gathered responses through both electronic and printed paper questionnaires. The survey consisted of background information questions, multiple-choice questions, and open-ended questions. It was conducted between 19 March and 31 March, 2023, together with the Union for Senior Services (VALLI). A study was conducted using a qualitative analysis of responses to open-ended questions.

### Participants and recruitment

The study population comprised Finnish individuals aged 75 and over from across Finland. The Ministry of Social Affairs and Health in Finland defines older individuals as those aged 75 years and older [27]. In Europe, where the statutory pension age ranges from 60 to 67 years [28], there is a growing demand for employees with basic digital skills across all sectors and working adults have encountered varying levels of digitalization in their jobs [29]. Since the age of 75 roughly coincides with about a decade into retirement, this age group has become progressively distanced from the digital transformation of the workforce and has not encountered work-related digitalization as extensively as younger adults [4].

VALLI recruited the participants via targeted emails sent to professionals working with older adults, volunteer workers in elderly care, Finnish elderly councils, and various pensioner and senior associations. These professionals and organizations distributed the questionnaire as an internet link, or in a downloadable PDF format for participants to fill out, or asked VALLI to send out the paper version. VALLI also sent an electronic newsletter specifically targeted at leaders of member organizations providing elderly services, and recruited some participants for the study during events for seniors.

### Survey instrument

The questionnaire was created by the research team with the help of an expert from VALLI (supplementary file 1). A previous survey on the digital inclusion of older adults conducted by VALLI in 2022 provided the basis for the questionnaire [30]. Some questions on healthcare and social services, and the electronic utilization of these services were also taken from the National FinSote Survey conducted by the Finnish Institute for Health and Welfare [31]. Older adults played an integral role in the testing and development phases of the digital inclusion survey. They participated in the development of the survey template, helping us adapt the digital terms and expressions and make them comprehensible for older adults.

In the initial section of the questionnaire, demographic information on age, gender, place of residence, and any potential constraints (such as sensory limitations) was gathered through multiple-choice questions, except for location, which was handled differently. Similarly, the second section’s questions on the utilization of smart devices and the internet were also presented in a multiple-choice format. In the third section, the questions on the participants’ experiences of digital health and social services were also predominantly multiple-choice. However, one question provided participants with the option to describe their experiences of using digital health and social services. The fourth section comprised three open-ended questions, allowing the participants to provide suggestions or express their preferences regarding the development of digital health and social services. Sections one to three are presented in other quantitative study [32], whereas this study utilized data from the open-ended questions in section four and information on demographics from section one. Given the diverse themes explored in the open-ended questions, and that they were distinct from the structured questions and constituted a substantial portion of the dataset (altogether 947 lines of data), they were analyzed separately from the other sections of the questionnaire for clarity and ease of interpretation. The number of open-ended responses (*n* = 601) was smaller compared to structured questions because the open-ended questions were optional, while the other questions were mandatory. Additionally, the length of the survey may have influenced the number of open-ended responses received. Quality control of the open-ended questions was ensured by having the research group, which included a gerontology expert from Valli, screen the questions. Additionally, older adults were involved in designing the questionnaire to ensure that the questions were clear and understandable. The open-ended questions were as follows:

- In what ways would you like digital healthcare and social services to be improved to better serve your needs and enable you to utilize them more effectively?
- What factors would support and facilitate your use of digital healthcare and social services?

### Data collection

The responses to the survey were anonymous. The electronic responses were automatically recorded in the Questback system, and the results were presented to the research team in Excel format. The paper version responses were manually entered into the Questback system by the research team.

### Data analysis

At the outset of the data analysis, we categorized the respondents on the basis of whether they utilized synchronized digital health and social services or did not engage with them at all. Respondents who used synchronous chat or remote appointments with healthcare and social service professionals were classed as users of synchronized services on the basis of their responses. We defined synchronized services as those used simultaneously by clients and health or social care professionals. These include remote home care services (15.1.7), remote health care consultations (15.1.14), and remote social care consultations (15.1.15) and remote social worker services (15.1.12) (supplementary file 1). The use of synchronized remote services in Finland has increased in recent years, particularly following the COVID-19 pandemic [33, 34]. These services have proven especially useful in specialized medical care [34].

The responses to the open-ended questions (*n* = 601) were examined using content analysis, a method applicable to diverse forms of unstructured or semi-structured data [35]. This approach aims to elucidate human experiences and perspectives of a research phenomenon. Both researchers who completed the analysis brought valuable clinical expertise from backgrounds in nursing or medicine, coupled with a deep understanding of geriatric care. Furthermore, they possessed prior experience in researching digital health and social services. Initiating the process, the primary author (EL) and another researcher (SL) thoroughly reviewed all the responses to the open-ended questions multiple times to develop an initial overview of the data and to ensure the quality of the responses. The attendance of two independent researchers ensured the dependability of the analysis. After the researchers were familiar with the data, the next step was to select the unit of analysis, for example, one word, sentence, meaning or theme [35]. In this study analytical unit selected was meanings, since they encourage the researcher to look at the data rather than in fragmented pieces, which can enhance the overall coherence and integration of the findings [35]. Consequently, the authors coded meanings that aligned with the research question using Atlas.ti. After the codes were recorded, the researchers divided the open codes into subcategories. Next, we recorded these subcategories as categories, and finally, the data allowing, we formed the main categories by comparing the categories. After each stage of analysis two researchers discussed about the analysis and discussed of any divergent opinions. Once the analysis was complete, a research group commented the formed categories and gave suggestions to improve them. The suggestions mainly concerned the naming of the categories.

### Ethical considerations

The study received approval from the steering committee of the research project, which comprised representatives from the Finnish Ministry of Social Affairs and Health and the Ministry of Finance. The survey itself was anonymous. The letter enclosed for the survey participants explained that the survey was part of a research project investigating the impacts of digital health and social services within the Finnish Government.

## Results

### Demographics of the respondents

We received 1124 completed questionnaires (1011 electronic and 113 paper) of which 1100 were fully completed and thus included in the analysis. Of the respondents, 64% were 75–79 years old and 68% were women (Table 1). The responses came from all over Finland, geographically from the southern regions to Lapland, and thus covered both small municipalities (< 1000 inhabitants) and large cities. Of the respondents, 22% were users of synchronized digital health and social services and 78% were not.

**Table 1:** Respondents’ demographics

Age (years)	Male	Female	No response
75–79	210	494	2
80–84	95	168	4
85–89	28	70	1
90–94	6	15	
95–99	1	6	
**Total**	**340**	**753**	**7**

### How can digital health and social services be designed to meet the needs of the older adults?

We identified eight main categories of older adults’ preferences regarding digital health and social services: (1) usability, service design, and security; (2) training, support, instructions, and information; (3) flexibility of compatible devices; (4) understandable language and interpretation of laboratory results; (5) available and accessible services; (6) desired functionalities; (7) delivery of information for viewing; and (8) personalization. Table 2 presents the main categories, generic categories, subcategories, and representative quotes.

**Table 2:** Main categories, subcategories, and representative quotes

Main category	Generic categories	Subcategories (total of 127 codes) / Has used synchronized DHS (number of codes)	Subcategories (total of 376 codes) / Has not used synchronized DHS (number of codes)
1. Need for user-centered design	Usability	Clarity (9)	Accessibility (20)
		Accessibility (8)	Discoverability (16)
		Discoverability (6)	Clarity (15)
		Clearer search (4)	Simplicity (12)
		Simplicity (2)	Clearer search (8)
		Clear logic (2)	Lighter login/authentication (5)
		Reliability (1)	Clearer logic (5)
		Authentication/clarity of authentication (1)	Reliability (4)
	Service design	Plain language (7)	Plain language (19)
		Consolidated information in one place (5)	Faster delivery of information (4)
		Displaying laboratory test reference values (4)	Consideration of senses (4)
		Inter-service transitions/integrations (3)	Less variability (3)
		Visual appeal (2)	Improved design (2)
		User consideration (3)	Availability of information (2)
		Minimizing unnecessary repetitions (2)	Displaying laboratory test reference values (2)
		Improved design (1)	Inter-service transitions/integrations (1)
		Consideration of senses (1)	More information about laboratory results (1)
		Only essential information (1)	
		More information about laboratory results (1)	
	Security	Security (1)	Security (8)
			Fear of being scammed (1)
2. Training, support, instructions, and information	Training and support	Support phone line (4)	Training (34)
		Digital support (3)	Digital support person (11)
			Digital support (10)
			Training at home (3)
			Multilingual support (1)
	Instructions	Instructions (1)	Instructions (19)
			Instruction videos (2)
	Informing of digital services	Informing (3)	Informing (8)
		Information on its use/purpose (2)	
	Professionals’ digital competence	-	Digital competence of healthcare and social service personnel (2) Training for healthcare and social service professionals in the use of digital tools (1)
3. Importance of functioning devices and connections	Devices	The challenges of acquiring and implementing devices (3)	The challenges of acquiring and implementing devices (23)
	Connections	Functional connections (8)	Functional connections (1)
4. Available and accessible digital services	Availability of digital services	Availability of digital services (1)	Availability of digital services (13)
	Faster and continuously available services	Faster contact (7) Quicker responses (4) Faster availability of services (1)	Faster contact (10) Quicker responses (10) Faster availability of services (9) 24/7 service (1)
	One-stop service	One-stop service (2)	One-stop service (3)
	The need for traditional services	Need for traditional services (7)	Need for traditional services (13)
5. Functionalities desired for digital services	Communicating to professional	Video connection (2) Contact opportunity (2)	Communication method similar to email (3) Sending pictures (2)
		Two-way messaging (2)	Feedback service (1)
		Submitting information to professionals (1)	Chat option (1)
		Chat option (1)	Submitting information to professionals (1)
	Functions that promote treatment	Possibility to schedule appointments (5) Renewing prescriptions (2) Alarm systems/reminders (1) Improved monitoring of health conditions (1)	Being able to schedule appointments (7) Alarm systems/reminders (2) Renewing prescriptions (1) Appointment availability via text message (1)
	Personalized service	Personalized instructions (1)	My own digital doctor/nurse (13)
		Better information (e.g., medication information) (1)	Personalized service for individuals (2) Handling small, simple matters (1)

#### Need for user-centered design

Participants indicated that there was a need for *user-centered design of digital services*, which included *usability*,* service design*,* and security. Usability* of digital health and social services was preferred amongst participants. Both segments, regardless of whether or not they had utilized synchronized services, appeared to prefer digital health and social services that were clear in different ways, assessable, discoverable, simple to use, and reliable. The respondents emphasized the need for clarity, clear search functions, and clear logic. In addition, the respondents wanted a simple or lighter authentication process that only required them to identify themselves once and did not require multiple codes to log in to the digital services. Several participants reported difficulties in searching for information, describing the service providers’ websites as complex, which hindered their ability to locate the information they sought. They also found the search functions and using keywords to be complicated, as they did not consistently yield the required results.

*“The fewer links there are for navigation on websites*,* the better. Sometimes*,* it takes a lot of clicking before the right page opens/you find the right page*,*” 75–69-year-old female who has used synchronized DHS.*

Thus, participants appeared to prefer the usability of digital services, where they could easily find what they were looking for and the authentication process was simple.

Several participants suggested that the digital service developers should consider the preferences and experiences of older adults when developing digital services. There was a consensus that *service design* — such as using plain language, enabling inter service transitions or integrations, incorporating visual elements, consolidating information in one place, and notifying user consideration, and potential sensory impairments — are essential.

Some participants found the language used in digital health and social services challenging. Some reported finding it difficult to understand the medical or bureaucratic terminology, whereas others found the digital language and terminology to be complicated. A desire was also expressed for more comprehensive and easy-to-understand information, especially concerning laboratory results. The respondents wanted to be able to clearly see the reference values and for health professionals to give them more detailed interpretations to enhance their understanding.

*“I’d appreciate responses from doctors and nurses in ‘plain language’. Sometimes it’s difficult to understand what they mean*,*” 75–79-year-old female who has used synchronized DHS.*

From the service design point it was also important to emphasize, that the participants would have preferred having all necessary information in one single digital service. They explained that information was currently scattered across various digital health and social services. For instance, some healthcare and social service providers recorded the data on their own digital platforms, whereas other information was added to a nationally administered platform. The participants were sometimes dissatisfied with how long it took for data to be updated in the digital service.

*“I want consistent personnel*,* no repetition of the same discussions with different professionals who initiate similar conversations from scratch each time*,*” 75–79-year-old male who has not used synchronized DHS.*

The participants wished developers to better understand their point of view when developing digital health and social services.

*“When geeky boys create websites*,* they often fail to understand the position of slow or non-tech-savvy elderly individuals*,*” 80–84-year-old female who has not used synchronized DHS.*

From the developers, the older adults expressed a desire for larger pushbuttons on websites and applications. They also emphasized that digital health and social services should be designed in a way that eliminates unnecessary repetitions, such as when filling out online forms. The integrations of different digital services should be built in a way that ensures a smooth and simple transfer of patient-related information.

Some of the older adults had difficulties related to sensory impairments, such as hearing or visual disabilities. For instance, individuals with hearing difficulties expressed a desire for an option to contact healthcare and social service providers via email.

*“As a person with a hearing impairment*,* I would like to access all services via email or similar means*,*” 75–79-year-old female who has not used synchronized DHS.*

Finally, the participants wanted digital health and social services to prioritize *security* and they expressed fear or being scammed.

*“Almost everyone is afraid of being scammed when using electronic connections*,*” 75–79-year-old male who has not used synchronized DHS.*

Some participants, especially those who had not used synchronized digital services, felt somewhat insecure about using them, expressing concerns about hoaxes on digital platforms that may mimic actual digital health and social services.

#### Training, support, instructions, and information

The participants expressed a desire for *training* and *support* on how to use digital health and social services. Participants’ preferences for training and support varied based on their experience of synchronized digital services. Those who had not used synchronized digital services wished for more extensive training and support, while those with some experience wanted to be able to call someone and ask for help. Consequently, the wish for more training and support in using digital services or devices was shared, suggesting that training sessions could be organized in communal spaces or in the older adults’ homes. However, the need for support and training was emphasized much more by those who had not used digital health and social services.

*“It would give me confidence if I was sure that I understand the matter correctly. Training at a slower pace would help it stick better in my memory*,*” 80–84-year-old female who hasn’t used synchronized DHS.*

Some participants mentioned receiving support from children or family members for using digital health and social services, but it was acknowledged that not everyone had such support. Those who lacked experience in using digital services also wished for support from healthcare and social service personnel for navigating these systems. Especially participants wished support by phone or at home. It was also emphasized that the training should be provided in different languages, especially as Finland has two national languages. A desire was also expressed for better *instructions*, including access to online guidance videos, and paper instruction guides.

*“I would definitely like to have a PRINTED guidebook covering the possibilities discussed here. It could serve as a starting point*,* with a gradual transition to using online services more extensively*,*” 85–89-year-old female*,* who has not used synchronized DHS.*

*Informing of digital services* was perceived important from the perspective of the participants. It was perceived a need for more information from the digital health and social service providers, on what services they offered and the purposes for which digital service could be used.

*“First and foremost*,* there should be more communication to inform people of their (healthcare and social services) options*,*” 75–79-year-old female who has used synchronized DHS.*

Finally, some participants also emphasized the importance of healthcare and social service *professionals’ digital competence* and being able to obtain services through them.

#### Importance of functioning devices and connections

The participants highlighted the necessity of having appropriate *devices* to use digital health and social services. Many found some devices to be too expensive, and several reported using outdated devices that hindered their engagement with digital services.

*“Elderly individuals could indeed learn to use devices if they had the means to afford them*,*” 75–79-year-old-female who has used synchronized DHS.*

Some participants expressed a need for assistance in selecting and acquiring devices such as laptops, tablets, or smartphones, or suggested that they could be free of charge from public organizations. Moreover, difficulties obtaining suitable, functional internet *connections* further complicated their use of digital services.

#### Available and accessible digital services

Participants wished for available and assessable services entailing *availability of the digital services*, having *faster and continuously available services* that enable *one-stop service*. Participants expressed a desire for more digital health and social services, especially in public settings.

*“It’d be really good to have digital service*,* while you’re still in good enough condition to learn them. However*,* they’re not yet available in the public sector*,*” 75–79-year-old female who has not used synchronized DHS.*

Participants wanted digital services to facilitate rapid contact with professionals, allowing them to quickly and flexibly be able to seek advice on various healthcare and social service matters.

*“If only pressing a single button could enable you to contact the staff you need*,*” 75–79-year-old female who has used synchronized DHS.*

Some participants reported long delays in responses from digital services and wanted professionals to answer them more quickly to enhance the efficiency of the system. Participants also wished that the digital services would enable them to handle their affairs through a single point of access: one main gateway.

*“Following a single point of access*,* which centralizes all services behind a single main gateway*,*” 75–79-year-old male who has used synchronized DSHs.*

Finally, participants also highlighted the *need for traditional services*. Some older adults did not want all services to be transferred to the internet; traditional services are still needed because not all older adults are able or willing to use digital services and some healthcare and social service issues are too complex to be handled without face-to-face contact.

*“I am 83 years old*,* and I don’t approve of transferring the monitoring of the health status of the elderly to the internet*,*” 80–84-year-old female who has not used synchronized DHS.*

#### Functionalities desired for digital services

Participant wished for different functionalities from digital services, namely functionality *enabling communicating to professionals*, *functions that promote treatment*, and functionalities that enable more *personalized service*. As the study population of older adults spanned across Finland, the digital health and social services that were available varied in the different wellbeing services counties, that are responsible for organizing healthcare and social services in Finland. The participants wished for different options (e.g., chat, asynchronous messaging, email) for communicating with healthcare and social service professionals.

The participants also expressed a wish for functions that promote treatment. They wanted to be able to book appointments, send information such as measurements to professionals, and renew prescriptions. They also wanted options for online contact, access to alert systems, and to be better able to monitor their conditions through digital health and social services.

*“Despite hearing aids*,* it can be very challenging to speak on the phone at times. For instance*,* in situations where there is an urgent need to make a call*,* and my husband is not present to assist. It would be beneficial to have an email-like communication method as well*,*” 75–79-year-old female who has not used synchronized DHS.*

The respondents wanted *personalized digital health and social services*, and to engage with familiar physicians, nurses, or other professionals through these platforms. They sought personalized guidance tailored to their specific situations instead of generic information and preferred human communication over artificial intelligence. The older adults acknowledged that digital service was suitable for addressing simple health issues but emphasized the importance of consistent care personnel and maintaining a continuous care relationship, even via digital platforms.

*“At the moment*,* certain data is being directed to both Maisa and OmaKanta*,* while some information is exclusively channelled to one or the other*,*” 75–79-year-old male who has not used synchronized DHS.*

Some of the highly digitalized wellbeing services counties already offered these mentioned functions, but not all did.

## Discussion

This study aimed at comprehensively investigate the preferences and needs of older adults regarding the use and development of digital health and social services. Based on older people’s preferences, there are several ways to design digital health and social services to better meet their needs. Services should be designed with a user-centered approach, incorporating more training, support, instructions, and information. It’s also essential to ensure that devices and connections function properly, consider the availability and accessibility of digital health and social services, and include features that older individuals specifically want to use.

According to our findings, it is essential that digital health and social services are designed to align with the specific requirements of older adults. Several earlier studies support this finding [36, 37]. The older adults in this study also indicated a lack of adequate devices for utilizing digital services. Earlier studies [38, 39] also found this and lacking internet access to be a barrier to using digital health and social services. In the present study, the participants suggested that service providers could provide them with devices. This could reduce the “digital divide” among older adults who use and do not use digital services. It would be useful to investigate the cost-effectiveness of this solution in future research to find our whether providing digital devices by public funding could increase the use of digital services and decrease other health and social care expenses.

Raja et al. [4] proposed that future research investigating older adults’ preferences for digital services should consider their past experiences with these services. Our findings reveal distinct differences between the preferences of older adults who have engaged with synchronized digital health and social services and those who have not. We propose that recognizing older adults’ proficiency in utilizing digital services should be essential. This underscores the necessity for a more refined segmentation of services, tailored to the specific capabilities of this demographic. One possible solution would be to extend the Subjective Health Experience (SHE) model to the digital health and social services [40], as this model includes health experience, population characteristics, healthcare needs, and appropriate support.

Our findings indicate that older adults who had utilized synchronized services did not seem to express a requirement for that much training or additional support, whereas those who had not used such services expressed a need for training and other forms of support. This finding may facilitate the targeting of training in a more efficient manner. Previous technology use and education level were not primary drivers for adopting new digital solutions could encourage older individuals who hesitate due to lack of experience to explore telehealth and new devices without fear [4]. An earlier study showed that as much as 70% of older adults reported needing support for using digital services [13]. Based on our findings, various training methods and support approaches should be tailored accordingly. Older adults without prior experience in using digital services would benefit from more intensive training and support, while those familiar with digital services could benefit from lighter forms of assistance. In an ideal scenario, offering timely and genuinely targeted support could potentially increase the adoption of digital services among older adults. It might be beneficial to delve into digital capabilities, acceptance, and willingness. Understanding how older adults navigate and embrace digital technologies can further help the customization of services, ensuring that they align seamlessly with the varying degrees of digital proficiency and acceptance within this demographic. According to an earlier study, good digital competence may promote the use of digital services among older adults [16]. Expanding the user base of digital health and social services by providing older adults with more training is likely to enhance the overall effectiveness of digital services.

Some participants in our study suggested that healthcare and social service professionals could assist them with digital services. However, given how limited healthcare and social service resources currently are, this might be difficult [3]. Mielonen et al. [13] have suggested that close cooperation with third sector organizations might be a plausible solution for providing older individuals with more training and other forms of support. We recommend conducting additional studies to assess the cost-effectiveness of training in digital health and social services for specific segments of the older population, considering their digital literacy and other capabilities.

Many of the findings were related to the design of the digital health and social services: their language, functionalities, or views. To ensure that digital health and social services are effective and usable, it is crucial to involve the end-users, particularly older adults, in their development process. It has also been suggested that the active engagement of end-users is paramount in the development of human-centered solutions. As many services are now becoming digital, designing them to enable value co-creation between a service provider and customers has become an increasingly important issue [41]. Peng et al. [42] argue that value co-creation is needed in healthcare. This is in line with the current trend of more personalized healthcare, especially in digital services. When developing digital health and social services, it is crucial to engage individuals of various age groups in co-designing them to guarantee that the solutions are effectively customized for the specific needs of older adults. Moreover, both value co-creation and value co-destruction perspective should be addressed when services are being designed, to ensure interaction between older adults and professionals. Further research could use the ladder interview technique, which is based on the Personal Construct Theory (PCT), to more thoroughly investigate which values and goals drive people’s use of technology [41].

Not only is the usability of digital health and social services crucial, but also their integration into service offerings and alignment with service promises. Our study, along with an earlier review [39], highlighted that older adults are not always familiar with the digital service offerings, which can hinder their usage. An earlier review suggests that healthcare providers should actively promote and advertise digital services that may be of interest to older adults, as they may not be aware of what is available [39]. In our study older adults expected prompt responses and the ability to communicate with familiar health or social care professionals, which digital solutions do not always facilitate. Additionally, a recent review found that older adults view interpersonal relationships with clinicians as a facilitator for adopting communicative digital services, as trust has already been established before transitioning to online communication [39]. Therefore, digital health and social services should be evaluated as part of holistic processes and inspected through the lenses of continuity of care, rather than solely focusing on their usability. This approach might enhance older adults’ willingness to use digital services if they can communicate with familiar professionals, receive prompt responses, and have a clear understanding of the available digital service offerings.

Although the research questions covered both healthcare and social services, the responses of the older adults mostly concerned digital health services, such as assessing own health-related information or viewing and reviewing prescriptions. In Finland, where the study was conducted, healthcare and social services are integrated [3], but healthcare services are more digitalized. Finland is not unique in implementing integrated approaches: the United Kingdom [43], Sweden [44], and Norway [45] also have somewhat integrated healthcare systems. A recent umbrella review by Härkönen et al. [46] highlighted the absence of research on the effectiveness of digital social services. Thus, we suggest further research on digital social services from different perspectives to gain more understanding of them.

### Limitations

Study participants were recruited both via email and in person at various events for elderly individuals, where respondents had the option to complete a printed paper questionnaire. However, the majority of responses were submitted through the electronic questionnaire, which may have led to a sample that overrepresents more digitally competent participants. The survey was not sent by mail to all Finnish individuals aged over 75; instead, participants learned about the study through email, elderly care volunteers or workers, different senior organizations, or by attending events.

The survey targeted individuals aged 75 and over, a demographic that may exhibit conditions such as dementia, which potentially introduces recall bias. To minimize bias in wording, the survey questions were crafted with input from a professional from VALLI, who was experienced in designing surveys tailored to elderly individuals. Elderly individuals also participated in the planning and development of the survey template. One limitation of the study is the brief response period of the survey, which was approximately two weeks. If the response period had been longer, we may have reached more participants. Finally, our survey was limited to Finnish-speaking population which may have excluded some older adults using Swedish as native language.

## Conclusions

In conclusion, it is essential to consider the digital capabilities and preferences of older adults when developing and designing digital health and social services. They should be actively involved from the early stages of the development of strategies concerning digital health and social services, using value co-creation and segmentation as tools and avoiding value co-destruction. Segmenting users, potential users, and non-users of digital health and social services and recognizing the various segments that need improvement can facilitate the design of digital services and the provision of other healthcare and social services. Finally, digital services should be examined more broadly as integral components of service processes, as some preferences of older adults focus more on the development of processes rather than the digital services themselves. Additional research is also warranted on the cost-effectiveness of segmentation-based training or the provision of adequate digital devices for older adults to increase the adoption of digital health and social services among this demographic.

## Electronic supplementary material

Below is the link to the electronic supplementary material.

Supplementary Material 1

Supplementary Material 2

## Data Availability

The datasets generated and analysed during the current study are not publicly available, but they are available from the corresponding author on reasonable request.
