# Supplementary material for: Older adults’ preferences in the utilization of digital health and social services: a qualitative analysis of responses to open-ended questions
Source: BMC Health Serv Res. 2024 Oct 4;24:1184. doi: 10.1186/s12913-024-11564-1 (PMC11451244; doi:10.1186/s12913-024-11564-1)
Supplement: Supplementary file 1 — Supplementary Material 1 [file 12913_2024_11564_MOESM1_ESM.docx]

**Experiences with digital social and healthcare services**

This survey aims to investigate the usage and experiences of digital social and healthcare services among citizens aged over 75. The survey is conducted as part of a project examining the impacts of digital services in social and healthcare, initiated by the Finnish Government.

Digital social and healthcare services refer to services in the fields of social and healthcare that are accessed via the internet or mobile devices, such as Omakanta, Maisa, Omaolo, or electronic appointment scheduling systems.

**A. Background information**

1a.Your age

75–79 years 80–84 years

85–89 years 90–94 years 95–99 years 100 or over

2a. Your gender

female male other I don’t want to tell

3a. Your place of residence

­­­­­­­­­­­­­­___________________________

4a. I have some limitation (e.g., vision, hearing, mobility impairments, tremors, financial constraints) that restricts the use of digital services (answer options: yes / no). If you answer no, please proceed to the next question 1b.

If you answered **yes**, what type of limitation do you have?

__ a sensory impairment, such as visual or hearing impairment

__ a mobility restriction or disability that prevents the use of digital devices

__ my financial situation limits the acquisition/maintenance of digital devices

**B. Use of Smart Devices and the Internet**

1b. Do you use digital social and healthcare services (e.g., Omakanta, Maisa, electronic services)? (answer options: yes / no) If you answered yes, proceed to question 2b. If you answered no, please respond to the following question, but do not continue to fill out the survey any further / the survey ends in Questback.

If you answered no, what is the reason for not using digital social and healthcare services? (check the appropriate box)

- I do not know how to use digital services
- I find the use of digital services difficult
- I find the use of digital devices difficult
- I do not have access to telecommunications and/or digital devices
- I do not understand the language used in digital services
- Digital services are generally not available in my native language
- I am unable to authenticate myself for digital services
- I am afraid to use digital services because I do not trust them or fear being deceived
- I do not want to use digital services at all.
- Some other reason, what? _____________________________________________

If you answered no to question 1b, please do not continue filling out the survey any further / the survey ends in Questback.

2b. Do you use the internet? (answer options: I use it myself / I use it with assistance from another person or someone else uses it on my behalf / I do not use it)

- For online transactions
- For obtaining information

3b. Assess your proficiency in using the internet. (check the appropriate box yes/no)

- I can open the desired webpage
- I can connect my device to the internet
- Selecting search terms is easy for me when searching for information on the internet
- I can fill out an electronic form (e.g., tax return, passport application)
- I know how to download applications to a mobile device

4b. Assess your cybersecurity skills. (check the appropriate box yes/no)

- I can identify cybersecurity risks (e.g., using the same password in multiple places)
- I can recognize phishing attempts (e.g., unexpected email about winning the lottery or disguised as investment advice)
- I can search for reliable health information

**C. Experiences with digital social and healthcare services**

1c. What is your opinion on the following statements regarding electronic services? (answer options: strongly agree - strongly disagree, 5 levels)

- A personal meeting cannot be replaced by electronic contact.
- The services I need are not available electronically.
- I am concerned about the security of my personal information.
- Telecommunications connections are too weak in my area.
- I need guidance on how to use social and healthcare online services.

2c. What is your opinion on the following statements regarding the benefits of social and healthcare electronic services? If you cannot assess electronic services, select the option "neither agree nor disagree" (answer options: strongly agree - strongly disagree, 5 levels)

- Help to maintain healthy lifestyles
- Help assess the need for services
- Facilitate finding and choosing services that suit me
- Facilitate service use regardless of time and place
- Facilitate collaboration with professionals
- Help take an active role in managing my own health and well-being
- Help tailor the service to my individual needs
- Help care for the health, well-being, or functional capacity of a loved one
- Save me time and expedite service delivery
- The professionals I interact with electronically are competent
- Interaction with healthcare professionals electronically feels natural

3c. How many times have you conducted an appointment electronically (e.g., via video call or chat) with the following professionals in the past 12 months? (answer options: never / once / more than once)

- Doctor
- Nurse
- Social worker or social counselor
- Other social or healthcare professional

4c. If you wish, briefly describe your experiences with electronic appointments with healthcare professionals (optional):

_________________________________________________________________________

5c. Have you used the following electronic social or healthcare services in the past 12 months?

If yes, rate the quality of the service with a grade (4-10) (answer options: yes/no - grade)

- Omakanta (e.g., own prescriptions and health information)
- Omaolo
- Health care symptom assessments
- Well-being check-ups
- Terveyskylä.fi (e.g., Diabetes House and Mental Health House)
- Electronic service of your municipality or region (e.g., Hyvis.fi, Maisa, Miunpalvelut, NettiRassi, Oulun omahoito or Virtu.fi)
- Electronic service of your occupational health care
- Remote home care services
- Kela's services
- Electronic social welfare services
- Social counseling chat service
- Senior Info's electronic services
- Electronic social welfare applications (e.g., livelihood support application)
- Electronic contact with a social worker
- Other electronic social welfare service
- Remote consultation in healthcare with a doctor or nurse
- Remote consultation in social services with a social counselor or worker

6c. Have you done the following activities using a mobile device, computer, or smart technology in the past 12 months? (answer options: no / no, but I would be interested / yes)

- Searched for information on promoting your own health and well-being about illnesses, their symptoms, or treatment
- Taken risk assessments, symptom assessments, health check-ups, or written assessments of your own functional capacity
- Conducted a well-being check-up
- Searched for information about health or social services in your area
- Made an appointment for social or healthcare services
- Applied for social services or livelihood support
- Requested renewal of a prescription
- Used a smart safety bracelet, automatic assistance alarm device, or other smart technology supporting independent living
- Reviewed patient or client information recorded by professionals about yourself
- Received the results of laboratory tests or other examinations
- Received your own care or service plan
- Submitted your own measurement results or other customer-related information to professionals
- Received instructions from professionals based on, for example, test results or submitted monitoring data
- Used an application that supports monitoring or treatment of illness or symptoms
- Collected your own health data (e.g., blood pressure, pulse) into some electronic device?

**D. Development Suggestions and Requests**

If you wish, you can also respond to the following open-ended questions, aimed at gathering users' suggestions for the development of digital social and healthcare services.

1d. How would you like digital social and healthcare services to be developed to better serve your needs and enable you to utilize them more?

2d. What types of social or health issues would you prefer to address using technology?

3d. What factors would support and facilitate your use of digital social and healthcare services?

Thank you for your response!
